# Extreme Heterogeneity in Parasitism Despite Low Population Genetic Structure among Monarch Butterflies Inhabiting the Hawaiian Islands

**DOI:** 10.1371/journal.pone.0100061

**Published:** 2014-06-13

**Authors:** Amanda A. Pierce, Jacobus C. de Roode, Sonia Altizer, Rebecca A. Bartel

**Affiliations:** 1 Biology Department, Emory University, Atlanta, Georgia, United States of America; 2 Odum School of Ecology, University of Georgia, Athens, Georgia, United States of America; 3 Red Wolf Recovery Program, United States Fish and Wildlife Service, Manteo, North Carolina, United States of America; University of California, Berkeley, United States of America

## Abstract

Host movement and spatial structure can strongly influence the ecology and evolution of infectious diseases, with limited host movement potentially leading to high spatial heterogeneity in infection. Monarch butterflies (*Danaus plexippus*) are best known for undertaking a spectacular long-distance migration in eastern North America; however, they also form non-migratory populations that breed year-round in milder climates such as Hawaii and other tropical locations. Prior work showed an inverse relationship between monarch migratory propensity and the prevalence of the protozoan parasite, *Ophryocystis elektroscirrha*. Here, we sampled monarchs from replicate sites within each of four Hawaiian Islands to ask whether these populations show consistently high prevalence of the protozoan parasite as seen for monarchs from several other non-migratory populations. Counter to our predictions, we observed striking spatial heterogeneity in parasite prevalence, with infection rates per site ranging from 4–85%. We next used microsatellite markers to ask whether the observed variation in infection might be explained by limited host movement and spatial sub-structuring among sites. Our results showed that monarchs across the Hawaiian Islands form one admixed population, supporting high gene flow among sites. Moreover, measures of individual-level genetic diversity did not predict host infection status, as might be expected if more inbred hosts harbored higher parasite loads. These results suggest that other factors such as landscape-level environmental variation or colonization-extinction processes might instead cause the extreme heterogeneity in monarch butterfly infection observed here.

## Introduction

Much work during the past two decades has focused on understanding the spatial ecology of host-pathogen interactions. Some studies have shown that genetic variation in traits affecting host resistance and pathogen virulence can generate spatial variation in infection patterns [1]. Other work demonstrated that landscape-level heterogeneity in factors such as habitat quality, the relative abundance of host species, and geographic features such as rivers and mountains, can affect the spatial spread and prevalence of pathogens [2]–[4]. Understanding the pattern of spatial heterogeneity in infection is crucial for identifying key drivers of pathogen persistence and for predicting and managing disease risk.

Host dispersal patterns can have important consequences for spatial processes and the ecology and evolution of host-pathogen interactions [2], [5]–[8]. Some studies have shown that host movement among patches can facilitate pathogen persistence at the landscape level [9]–[11]. On the other hand, directed seasonal movement (i.e., long distance migration) can lower parasite transmission by allowing hosts to escape from parasitized locations [10], as has been suggested for warble flies affecting reindeer [12], and protozoan parasites infecting monarch butterflies [13]. Movement can further result in gene flow and the spread of host resistance alleles across a landscape, with studies of anther-smut in plants and viruses in moths showing that limited host movement or gene flow can generate high spatial heterogeneity in prevalence, allowing some patches to become heavily infected while others remain disease-free [14]–[16].

Here, we examined spatial heterogeneity in the occurrence of an obligate protozoan parasite (*Ophryocystis elektroscirrha*, hereafter called *OE*) infecting monarch butterflies (*Danaus plexippus*) on the island chain of Hawaii. Monarchs inhabit islands and continents worldwide and occupy a subset of the range of their larval milkweed host plants [17]. Monarchs are best known for undertaking a spectacular long-distance migration (up to 5000 km roundtrip) in eastern North America [18], [19], but they also form non-migratory populations that breed year-round in tropical and subtropical locations such as the Caribbean Islands, Central America and Hawaii. Monarchs colonized Hawaii and other Pacific Islands in the mid-1800 s [20], [21] following the introduction of their host plants, and now occupy most of the eight Hawaiian islands [17]. Monarchs in Hawaii breed year-round in habitats containing introduced larval host plants, especially *Asclepias physocarpa, Calotropis gigantea,* and *C. procera*. Hawaiian monarchs are smaller than North American migratory monarchs [22], and microsatellite markers showed that Hawaiian monarchs are genetically distinct from those in North America and New Zealand [23].

All monarch populations examined to date are parasitized by *OE*, and prevalence varies widely among regions [24]. Prevalence reaches the highest levels in monarch populations that breed year-round (e.g., South Florida, Cuba) and is much lower in populations that migrate long distances [25]–[27]. In particular, non-migratory monarchs likely experience higher rates of transmission due to continuous breeding activity and extended use of the same host plants for egg deposition [10], [28], as parasites are transmitted when infected adults scatter spores onto milkweed leaves [25], [29]. Larvae ingest the spores, parasites replicate internally, and adults emerge with millions of dormant spores on the outsides of their bodies [25], [30]. While no further parasite replication occurs at the monarch adult stage, infected adults suffer from decreased body size, eclosion success, lifespan, flight performance and migration success [13], [31], [32].

In this study, we sampled monarchs and recorded *OE* infection across replicate sites within each of four Hawaiian Islands over multiple years. Based on previously documented associations between monarch migratory ecology and parasite prevalence, we expected that *OE* prevalence would reach high levels across all sites sampled owing to year-round breeding and the limited potential for long-distance movement among monarchs inhabiting these oceanic islands. Because our field analysis showed extreme heterogeneity in *OE* prevalence within and among islands (and lower than expected prevalence overall), we further used neutral genetic (microsatellite) markers to examine evidence for host population structure. In particular, we asked whether genetic evidence indicates that host movement within and among islands might be limited, such that between-site variation in prevalence could be attributed to locally structured host sub-populations that are isolated from other patches. Finally, we asked whether measures of host neutral diversity (as indicators of genome-wide heterozygosity) might correlate negatively with parasite infection probability at the individual or patch level, as suggested by prior work in Soay sheep, sea lions and several other species, whereby animals with greater genome-wide diversity can better resist parasite infections than more inbred hosts [33]–[36].

## Materials and Methods

### Field Sampling

**Figure 1:**
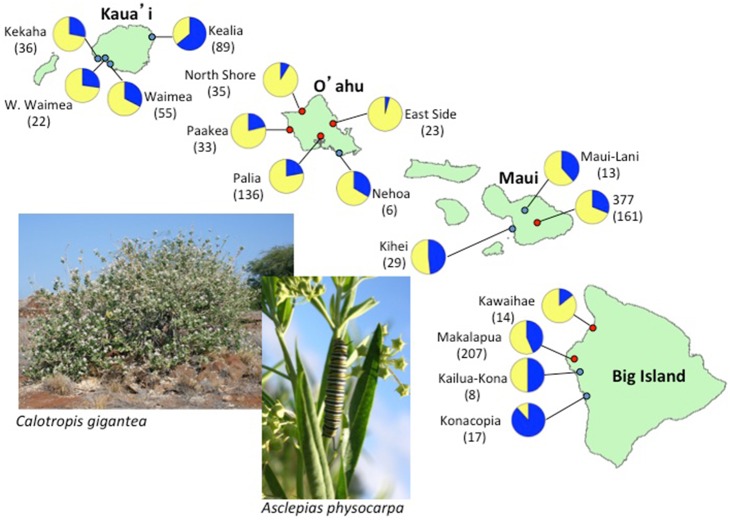
Variation in parasite prevalence on four islands of Hawaii based on field sampling from 2007–2010. Dark shading indicates the proportion of monarch infected with *OE* within subpopulations. Sample sizes are indicated within parentheses. Red dots indicate sites from which samples were further analyzed for microsatellite markers (Table S2). Photographs show two representative host plant species common throughout most islands.

We sampled monarchs and their parasites once per year in each of three years (2007, 2009, 2010) across four islands in Hawaii: Hawaii (Big Island), Oahu, Maui, and Kauai (Figure 1; Table 1). These islands differ in their total area and human population density. On each island, we identified 3–5 representative habitat patches where monarchs and their milkweed host plants (*Calotropis* or *Asclepias spp*.) occur (Table S1). Sites were separated by a minimum of 5 km and early site visits indicated that monarch adults and larvae were concentrated in host plant patches, as has been shown before for monarchs [37]. Field surveys occurred during the rainy season (Jan–Feb), and with each progressive year, we identified additional sampling sites (Table 1). In 2007, only the Big Island and Oahu were visited and sampled (N = 117 monarchs, 3 sites). In 2009, we expanded field efforts to Kauai and Maui and included more sites on the Big Island and Oahu (N = 388 monarchs, 10 sites), and in 2010, we added sites on all islands (N = 380, 15 sites).

**Table 1:** Monarchs sampled in Hawaii by collection site and year, with sample sizes (count) and the proportion of monarchs heavily infected with *O. elektroscirrha*.

	2007		2009		2010		Total Count	Total Proportion Infected
	Count	Average Proportion Infected	Count	Average Proportion Infected	Count	Average Proportion Infected		
Big Island	65	0.48	100	0.36	81	0.54	246	0.45
Kailua-Kona					8	0.50	8	0.50
** Kawaihae**					14	0.14	14	0.14
Konacopia Farms					17	0.88	17	0.88
** Makalapua**	65	0.48	100	0.36	42	0.55	207	0.43
Kauai			102	0.45	101	0.45	203	0.45
Kealia Beach			43	0.81	46	0.48	89	0.64
Kekaha Beach			26	0.23	11	0.36	37	0.27
Waimea			22	0.18	33	0.42	55	0.33
West Waimea			11	0.09	11	0.45	22	0.27
Maui			106	0.20	97	0.49	203	0.34
Kihei					29	0.48	29	0.48
** Maui 377**			100	0.20	61	0.49	161	0.31
Maui-Lani			6	0.17	7	0.57	13	0.38
Oahu	52	0.13	80	0.21	101	0.19	233	0.18
** East Side**					23	0.04	23	0.04
Nehoa St.					6	0.33	6	0.33
** North Shore**			5	0.00	30	0.10	35	0.09
** Paakea Rd**.	21	0.24	12	0.17			33	0.21
** Palai St.**	31	0.06	63	0.24	42	0.31	136	0.22
**Grand Total**	**117**	**0.32**	**388**	**0.31**	**380**	**0.41**	**885**	**0.35**

Sites in boldface were also examined for microsatellite markers (Table S3).

The field collections for this project did not involve endangered or protected species. We collected at three different private sites (Palia, Nehoa, and Konacopia) after receiving permission from S. and A. Montgomery, S. Marques, and E. Kilpatrick. The remaining collection sites consisted of roadsides, parks, or unprotected areas. No permits were necessary to collect these monarchs in Hawaii (collecting non-endangered butterflies in public areas is not prohibited in the United States, and monarchs themselves are not native to the Hawaiian Islands). All butterflies were transported to the University of Georgia, Athens, GA, under permission from the United States Department of Agriculture (USDA PPQ-526 Permit #11-04112 and Permit #06-01690 to S. Altizer).

Adult monarchs were captured using an aerial net between 0900 and 1600 hr. Following capture, monarchs were stored individually in glassine envelopes and held at 14°C for up to 6 hr prior to sampling. We recorded sex and forewing length to the nearest 0.01 mm. Wing condition, which qualitatively reflects age or distance traveled, was recorded in two ways. First, we recorded wing damage on a 0–4 scale, based on the number of wings with evidence of tears or other physical damage as might be caused by predators or contact with hard surfaces. Second, we recorded wing wear on an ordinal scale of 1–5, based on the level of scale loss (from newly emerged to nearly transparent wings) following Cockrell et al. [38].

### Measuring Parasite Prevalence and Transmission

Adult monarchs captured at each site were scored for parasite infection status based on the number of *OE* spores transferred to a 2.5 cm-diameter transparent sticker pressed against adult abdomens (described in Altizer et al. [26]). Samples were examined at 63X magnification to record infection scores on a 0–5 scale. This method is highly sensitive and past work showed that categorical scores are highly correlated with Log_10_ of quantitative spore loads [39] measured using an agitation and hemocytometer counting chamber method as described in Leong et al. [25] and Altizer et al. [26]. Samples with more than 100 spores were considered heavily infected; this classification includes the two highest spore load categories defined by Altizer et al. [26]. Importantly, heavily infected monarchs are those with infections likely caused by the ingestion of one or more spores as larvae, thus resulting in these individuals experiencing negative consequences of within-host replication [30]. In contrast, lower spore numbers can result from passive transfer of spores between adult butterflies [28], [30], [39]; these dormant spores cannot directly infect adults and must be ingested by a larva to cause a new infection. Following scoring infection status, we released the majority of monarchs at the collection site and kept a subset for genetic analysis (Table S2).

### Microsatellite Analyses

We used polymorphic microsatellite markers to determine whether monarchs were genetically differentiated between sites, or whether extensive gene flow occurs. Microsatellite marker development and PCR protocol were as described in Lyons et al [23]. Briefly, DNA for PCR was extracted from a 0.5 mm section of butterfly abdomen (male butterflies) or thorax (female butterflies) using the UltraClean DNA Isolation Kit from Mo-Bio (Carlsbad, CA, USA) and quantified using a Nanodrop 2000. We did not use female abdominal tissue as this could possibly contain DNA from male sperm. PCR was carried out in 15 µl multiplex reactions using the Type-It Microsatellite PCR kit (Qiagen). Only a subset of monarchs scored for infection status were collected for genetic work, so sites with nine or more samples were chosen to genotype (Table S2). In total, we genotyped 42 butterflies from two sites on the Big Island (Kawaiahea, N = 9; Makalapua, N = 33), 48 from four sites on Oahu (East Side, N = 9; North Shore, N = 9; Paakea, N = 11; Palia, N = 19), and 9 from one site on Maui (Maui377, N = 9) for 16 microsatellite loci (Table S3).

### Analysis of Field and Genetic Data

For field-collected samples, we used logistic regression (IBM SPSS Statistics 20.0) to examine the main effects of year, island, and site (as a random effect, nested within island) on variation in monarch infection status (at the individual level) as a binomial variable. We also included the island*year interaction effect, and individual-level predictors of sex, forewing length, wing damage and wing wear in the full model. Prior to analysis we excluded data from sites for which fewer than 5 samples were available. In a separate analysis, we investigated whether site-level variation in patch size, land use type (categorized as urban, suburban or rural), and host plant species explained variation in average prevalence measures (with details provided in Supporting Information).

To investigate host genetic differentiation, we used the software Arlequin 3.5.1.2 [40] to calculate observed and expected heterozygosity at each microsatellite locus for each site. We also used Arlequin to calculate deviations from Hardy-Weinberg equilibrium for each locus at each site, and used a sequential Bonferroni correction [41] to determine whether observed and expected heterozygosity levels were significantly different (α = 0.05). We excluded locus 137, which was not polymorphic or in Hardy-Weinberg in at least 5 out of 7 populations; the remaining 15 loci were used in subsequent analyses (Table S4).

Samples for each site were resampled with replacement using Poptools [42] to standardize sample size across sites for comparison of relative levels of genetic diversity. To do this, we calculated genetic diversity (using the value 1-Qinter) using Genepop version 4.1.0 [43] and allelic richness using ADZE-1.0 [44], which utilizes a rarefaction approach to account for differences in sample size. To understand the relative magnitude of within- and between-population genetic diversity, we carried out a locus by locus analysis of molecular variance using 10,000 permutations in Arlequin 3.5.1.2 [40] for six of the sites (Kawaihae, Big Island; Makalapua, Big Island; East Side, Oahu; North Shore, Oahu; Paakea, Oahu; Palia, Oahu). In this analysis, we combined sites based on island, and compared this to the variation among populations within groups (i.e. variation among sites within the same island) as well as genetic variation within sites.

We used the software STRUCTURE version 2.3.2.1 [45] to investigate population structure. We used an admixture model with uncorrelated allele frequencies to avoid the risk of overestimating the number of populations, K, and used the LOCPRIOR model to include location information for each butterfly. We did the latter to ensure that STRUCTURE would be able to detect subtle population structure. We also included 16 butterflies from New Zealand (Christchurch, Jan 2011) for comparison, as monarch populations in Hawaii and New Zealand were established within the last 170 years, and are thought to originate from North America through trans-Pacific dispersal [20], [21], [46]. Therefore, the inclusion of the New Zealand population allows us to determine that our markers are able to detect subtle and newly formed population structure.

We also examined population genetic structure using *F*_ST_ and *R*_ST_ statistics. These statistics are commonly used to calculate genetic differentiation, with levels of 0 indicating panmixia, and values higher than 0 indicating genetic differentiation. *R*_ST_ was developed as a more appropriate statistic for microsatellite markers, based on its use of a stepwise mutation model [47], rather than the infinite alleles model utilized in *F*_ST_ statistics [48]. Permutation tests (using 10,000 permutations), as implemented in Arlequin 3.5.1.2 [40] were used to determine whether pairwise *F*_ST_ and *R*_ST_ values were significantly different from 0. To further examine population genetic structure, we analyzed the correlation between site collection time and measures of genetic differentiation using Mantel tests implemented in the vegan library version 2.0-0 [49] in the statistical package R version 3.0.1. Finally, we calculated heterozygosity at the individual level by determining the proportion of heterozygous loci per butterfly. To investigate the effect of heterozygosity on infection status, we treated infection status as a binomial variable and performed a logistic regression using a generalized linear model (GLM with binomial error distribution, logit link) in R version 3.0.1.

## Results

### Parasite Prevalence and Transmission

**Figure 2:**
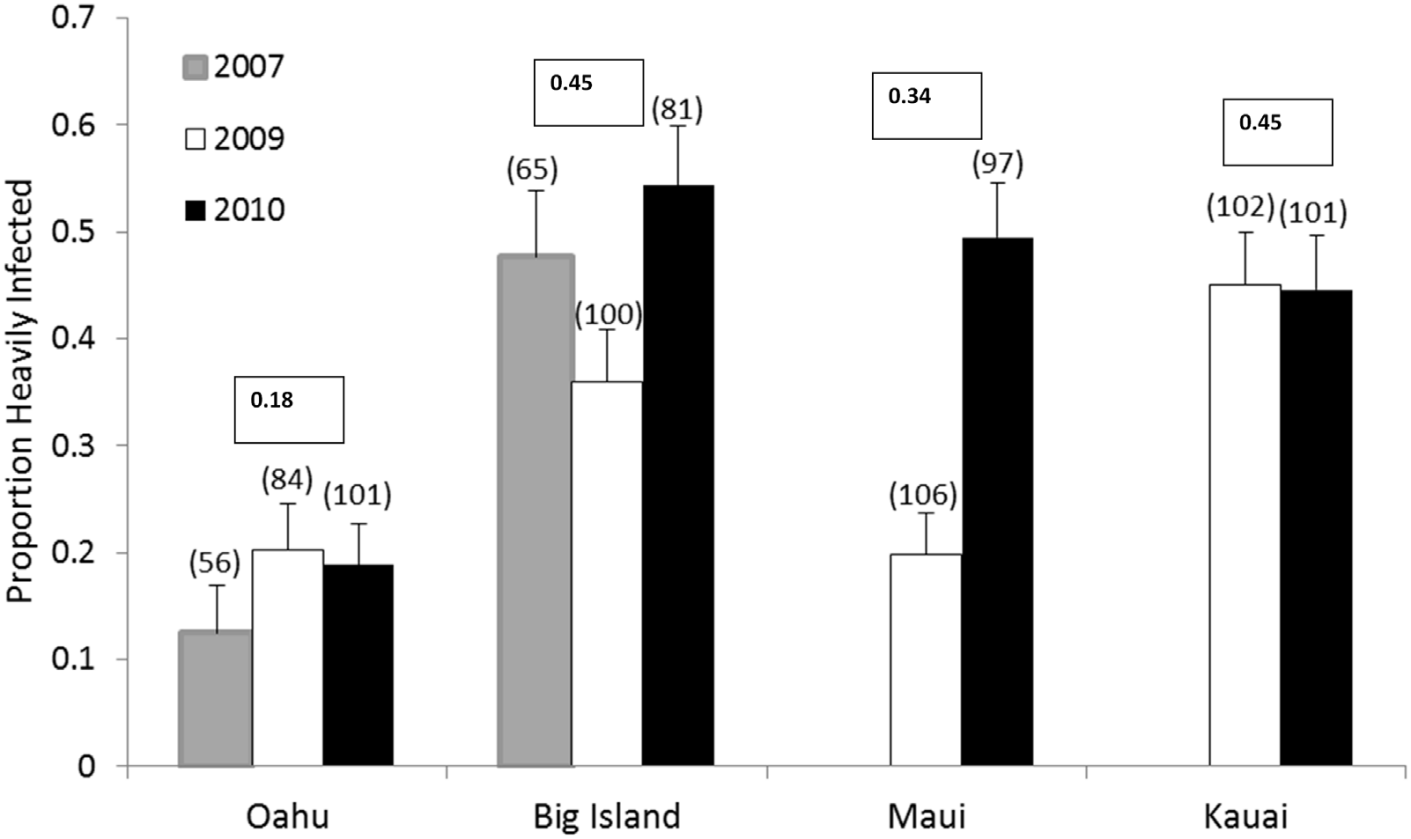
Proportion of monarchs heavily infected with *OE* for four Hawaiian Islands from 2007–2010. Sample sizes are in parentheses. Average prevalence per island over all sample years is shown in bold type. Field surveys in 2007 focused on the Big Island and Oahu only. Beginning in 2009, we sampled Maui and Kauai, and visited 3–5 sampling sites for each of the 4 islands. Sample sizes per island per year ranged from 56 to 105 (Table 1). Error bars indicate standard errors.

On average, 35.5% of monarchs were heavily infected with *OE* across all sites and years (N = 885; Table 1). We detected high variation in prevalence both within and among islands on the Hawaiian archipelago (Table 1; Figures 1–2), with the average proportion of heavily infected monarchs per site per year ranging from 0.00 to 0.88. Logistic regression showed a significant main effect of island on infection probability (Wald χ^2^ = 10.17, d.f. = 3, P = 0.017). In particular, the outer islands of Kauai and the Big Island showed the highest average infection levels (e.g., proportion of heavily infected monarchs on the Big Island, N = 246, and Kauai, N = 203, were both 0.45 when averaged across sites and years). By comparison, average prevalence was much lower on Oahu (proportion infected = 0.19, N = 233). Although we also observed a significant main effect of year (Wald χ^2^ = 16.13, d.f. = 2, P<0.001), with infection prevalence higher for 2010 than for 2007 or 2009 (Figure 2), differences in infection probability across islands were generally consistent among years (Table 1; Figure 2), as supported by a non-significant interaction between island and year (Wald χ^2^ = 5.20, d.f. = 4, P = 0.26).

At a finer scale, we detected strong within-island heterogeneity in the proportion of infected monarchs (Figure 1), and the effect of site nested within island was highly significant (Wald χ^2^ = 40.38, d.f. = 4, P<0.001). Of the 16 sites monitored through 2010, 9 were assessed for two or more consecutive years for monarch presence and parasite prevalence (Figure 2). Although some sites showed consistently low or high prevalence (Table 1), a separate simple linear regression analysis showed that *OE* prevalence per site in a given year was not predictive of prevalence the following year (R^2^ = 0.144, t_11_ = 1.30, P = 0.224).

Our multivariate logistic regression analysis further controlled for individual-level variables that might explain variation in *OE* infection. At the individual level, males (proportion infected = 0.49, N = 548) had higher infection prevalence than females (proportion infected = 0.44, N = 337) and this effect was highly significant (Wald χ^2^ = 16.0, d.f. = 1, P<0.001). Forewing length was negatively associated with infection status, such that infected monarchs had smaller wings than healthy butterflies (Wald χ^2^ = 9.95, d.f. = 1, P = 0.002). Wing wear (reflecting wing scale loss) also predicted variation in infection probability (Wald χ^2^ = 10.51, d.f. = 1, P = 0.001), such that infected monarchs were more likely to show greater wing scale loss. Wing damage (as an index of tatter), however, was not associated with monarch infection status (Wald χ^2^ = 0.32, d.f. = 1, P = 0.57).

A separate analyses of variance based on average prevalence by site and year (N = 28) showed that no site-level measurements (e.g., patch area, host plant species, habitat type) were significant predictors of variation in parasitism (results presented in Supporting Information). Although collection times (within the 0900–1600 hr range) varied among sites, there was no correlation between collection time and average infection prevalence (P = 0.52 for 2009, when detailed collection times were recorded).

### Neutral Genetic Variation and Population Structure

**Figure 3:**
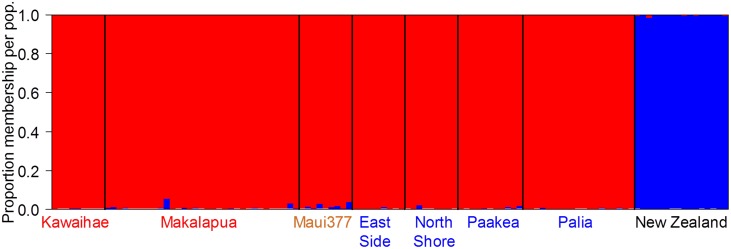
Structure plot showing that K (number of distinct populations)  = 2. Monarchs on the Hawaiian Islands form one admixed genetic population (red text = Big Island, orange text = Maui, blue text = Oahu). New Zealand monarchs are differentiated from Hawaii and form their own genetic group.

An AMOVA analysis using *R*_ST_ demonstrated that differences among sites, rather than among islands, are responsible for much of the observed variation in allele frequencies (Table 2). Therefore, our subsequent analyses were performed on the site scale. *F*_ST_ and *R*_ST_ analysis of site comparisons revealed moderate clustering based on island with the sites on Oahu differentiated from those on the Big Island (Table 3). The sites within Oahu were not significantly differentiated from one another except for one pairwise comparison (East Side and Palia). According to *F*_ST_ calculations, the Maui site was significantly different when compared to one of the Big Island sites (Kawaihae) and one of the Oahu sites (East Side). However, *R*_ST_ values for these comparisons were not significant. For thoroughness, we also looked at differentiation among islands and found similar results, with slight genetic differentiation detected between Oahu and the Big Island, as well as slight differentiation detected using *F*_ST_ between the Big Island and the other islands (Table S5). Thus, although there were some significant differences between sites, the observed levels of differentiation were low. This low level of genetic differentiation was confirmed with the analysis in STRUCTURE, which did not indicate any significant population structure (Figure 3). The lack of genetic structure is unlikely to be an artifact of our microsatellite markers as they clearly detect genetic differentiation between Hawaii and New Zealand butterflies. We also ran STRUCTURE without the inclusion of New Zealand, and still found a lack of population structure among the Hawaiian sites (Figure S1). We performed a Mantel test to determine whether genetic differentiation correlated with differences in site collection time and found that the relationship was not significant for *F*_ST_ (r = 0.04, P = 0.54) or *R*_ST_ (r = 0.22, P = 0.43). This lack of a correlation indicates that differences in collection times are not responsible for the small amount of genetic variation found.

**Table 2:** Results of analysis of molecular variance (AMOVA) comparing samples from locations within two islands (Kawaihae, Makalapua, East Side, North Shore, Paakea and Palia).

Source of variation	d.f.	Sum of squares	Variance components	Percentage variation	P-value
Among groups	1	1416.861 (17.408)	9.701 (0.134)	4.08 (3.66)	0.14467 (0.06940)
Among populationswithin groups	4	1636.393 (18.399)	7.752 (0.046)	3.26 (1.27)	0.01564* (0.12219)
Among individuals withinpopulations	174	38319.251 (603.755)	220.226 (3.470)	92.66 (95.07)	0.00000* (0.00000)*
Total	179	41372.506 (639.561)	237.679 (3.650)		

In this analysis, Kawaihae and Makalapua were grouped into the same group (Big Island) while East Side, North Shore, Paakea and Palia formed another group (Oahu). The analysis was carried out based on *R*_ST_ and *F*_ST_ values; results for the latter are shown in parentheses. Significant *P*-values, based on permutation tests in Arlequin v3.5.1.2, are indicated with asterisks.

**Table 3:** Pairwise *R*_ST_ and *F*_ST_ values between seven monarch buttterfly populations, as calculated in Arlequin v3.5.1.2.

	Kawaihae	Makalapua	Maui 377	East Side	North Shore	Paakea
**Makalapua**	*R*_ST_: 0.01886					
	*F*_ST_: 0.00524					
**Maui 377**	*R*_ST_: 0.02309	*R*_ST_: 0.01747				
	*F*_ST_: 0.06087*	*F*_ST_: 0.03410				
**East Side**	*R*_ST_: 0.07261*	*R*_ST_: 0.11108*	*R*_ST_: 0.04457			
	*F*_ST_: 0.03481*	*F*_ST_: 0.05370*	*F*_ST_: 0.06896*			
**North Shore**	*R*_ST_: 0.01119	*R*_ST_: 0.04047*	*R*_ST_: −0.01476	*R*_ST_: 0.03341		
	*F*_ST_: 0.04989*	*F*_ST_: 0.02963*	*F*_ST_: 0.00106	*F*_ST_: 0.02524		
**Paakea**	*R*_ST_: 0.11897*	*R*_ST_: 0.10292*	*R*_ST_: 0.00858	*R*_ST_: 0.03400	*R*_ST_: 0.00520	
	*F*_ST_: 0.08489*	*F*_ST_: 0.06238*	*F*_ST_: 0.01235	*F*_ST_: 0.03855	*F*_ST_: −0.00344	
**Palia**	*R*_ST_: 0.11233*	*R*_ST_: 0.03965*	*R*_ST_: 0.01722	*R*_ST_: 0.08108*	*R*_ST_: 0.03968	*R*_ST_: 0.04376
	*F*_ST_: 0.08310*	*F*_ST_: 0.03434*	*F*_ST_: 0.00090	*F*_ST_: 0.05444*	*F*_ST_: −0.01063	*F*_ST_: 0.00153

Asterisks denote values that are significantly different from zero. Note that all values are less than 0.12, and that significance is at the 0.05 level.

**Figure 4:**
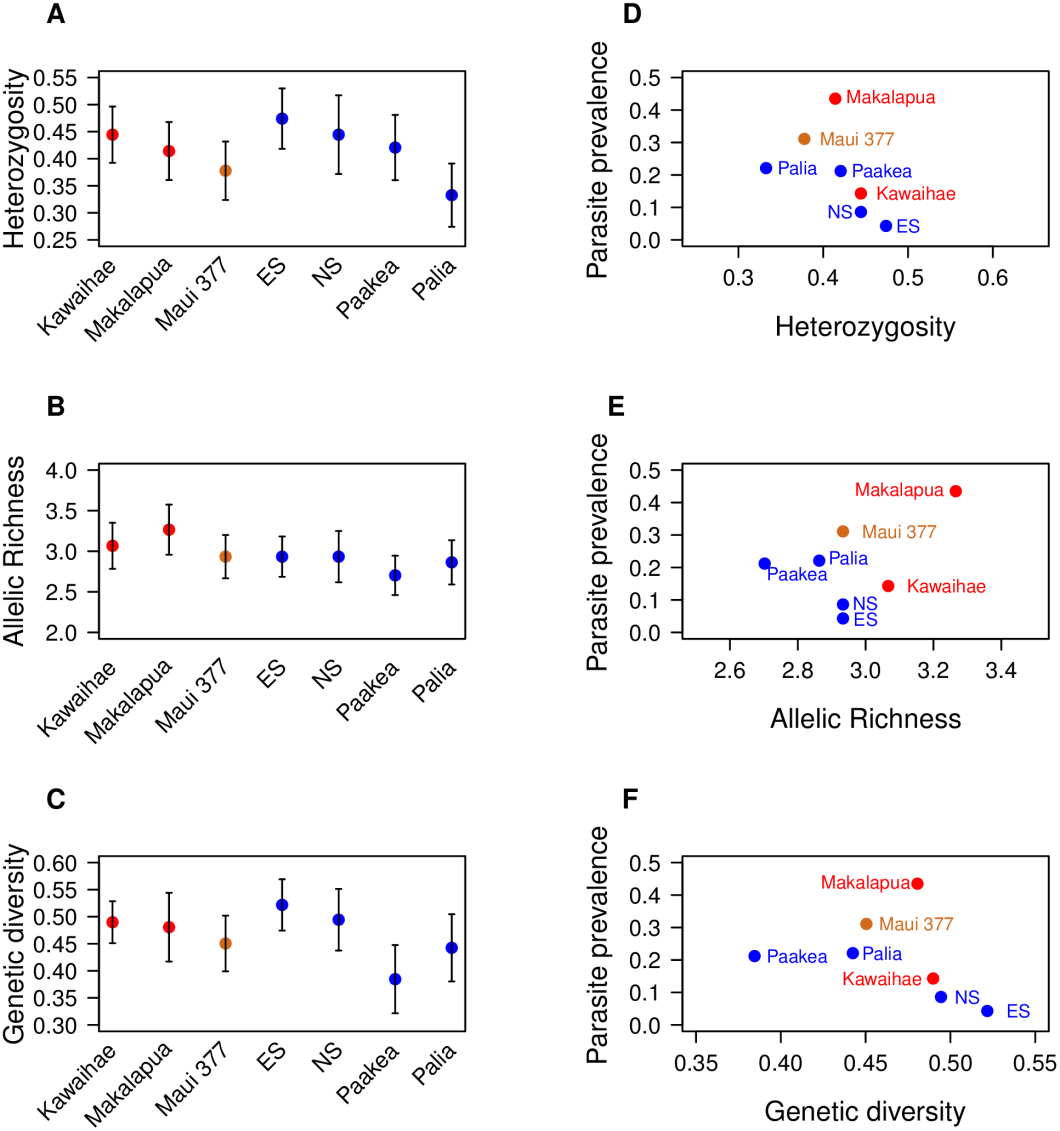
Measures of genetic diversity for monarchs from seven sites in Hawaii (red = Big Island, brown = Maui, blue = Oahu). ES refers to East Side and NS refers to North Shore, both of which are located on Oahu (**A**) Heterozygosity was found to be similar among the sites. (**B**) Allelic richness was similar amongst the sites. (**C**) Genetic diversity (using the value 1-Qinter, the inter-individual diversity within populations) was also found to be similar. (**D**) Heterozygosity did not correlate with parasite prevalence (r = −0.53; p = 0.22). (**E**) Allelic richness was not found to correlate with parasite prevalence (r = 0.46; p = 0.30). (**F**) Genetic diversity was not found to correlate with parasite prevalence (r = −0.36; p = 0.42). Error bars in panels A–C show ±1 SE across loci.

Mean heterozygosity levels among sites ranged from a low of 0.333 in Palia to a high of 0.474 in North Shore (both of these sites are within Oahu) and did not significantly differ among sites within islands (*F*_6,98_ = 0.65, P = 0.69; Figure 4A). Allelic richness ranged from a low of 2.702 (Paakea, on Oahu) to a high of 3.266 (Makalapua, on the Big Island) but did not significantly differ among sites (*F*_6,98_ = 0.44, P = 0.85; Figure 4B). Genetic diversity ranged from 0.385 in Palia to 0.522 in East Side (both of these sites are within Oahu) and did not differ significantly (*F*_6,98_ = 0.66, P = 0.68; Figure 4C).

### Associations between Genetic Diversity and Infection Status

No site-level measures of genetic diversity (mean heterozygosity, P = 0.22; allelic richness, P = 0.30; genetic diversity, P = 0.42) were found to correlate significantly with site-level averages of parasite prevalence (Figures 4D, 4E, 4F). At the individual level, average microsatellite heterozygosity was not found to predict infection status (χ^2^ = 126, d.f. = 1, P = 0.67).

## Discussion

Parasite prevalence was highly variable among and within the Hawaiian Islands. These results are unexpected, because the non-migratory status of monarchs of the Hawaiian Islands would lead us to predict that parasite prevalence should be relatively high across all sites. Instead, patterns identified here suggest that factors other than migratory behavior can play a major role in driving heterogeneity in parasite prevalence in this system.

In this paper, we examined whether population sub-structuring might be responsible for the among-site variation in parasite prevalence. In particular, limited host movement might allow for local inbreeding and the loss of genetic diversity in sites with small populations, which is known to increase pathogen susceptibility in other systems [33]–[36], and could more generally allow for the spatial segregation of host resistance alleles, leading to some sites with high resistance to infection and other sites with high susceptibility [14], [15]. Although we found slight to moderate genetic differentiation among sites and islands when using *R*_ST_ and *F*_ST_ statistics, we found no evidence of population structure using the program STRUCTURE. Moreover, parasite prevalence was not explained by variation in genetic diversity, heterozygosity, and allelic richness among sites. Thus, it appears that population genetic variation cannot explain the observed heterogeneity in parasite prevalence in this system. Instead, spatial environmental heterogeneity or ecological metapopulation processes might play stronger roles in determining infection heterogeneity in this host-parasite interaction.

Spatial variations in patch size, isolation and quality have been shown to alter parasite transmission and spatial spread in other host-pathogen systems. For example, empirical work demonstrated that landscape-level heterogeneity in habitat quality, host species diversity, and major geographic features such as water bodies affected the spatial spread and prevalence of pathogens ranging from rabies virus in raccoons to Lyme disease in white-footed mice [3], [4], [50], [51]. More generally, the dynamic structure of landscapes, particularly as a result of habitat fragmentation and other anthropogenic effects, can impact infectious diseases by affecting host species vital rates, density and distribution [52]. The Hawaiian Islands are known to vary in key ecological parameters such as total area, elevation, and human population density. Moreover, patches examined here differed in host plant species, patch size, and surrounding urban development. Although basic site level measurements collected here (Table S1) were not found to be correlated to infection prevalence, more comprehensive site-level data should be collected in the future, including actual numbers and distribution of host plants, elevation, temperature, precipitation, and monarch larval and adult densities. Host density in particular might correlate positively with parasite prevalence, as demonstrated by previous work on parasite infection in summer breeding North American monarchs [13].

Metapopulation ecology could offer a different perspective for understanding how spatial processes cause infection heterogeneity in the monarch-pathogen interaction [52]–[54]. Specifically, extinction and colonization processes across interconnected patches might generate spatial variation in prevalence (even in the absence of other environmental gradients) simply because sites differ in the timing of host and pathogen colonization [55], [56]. Here, we considered the possibility that patch age might predict infection probability, if older habitat patches are more likely to be colonized by the pathogen. One specific prediction might be that patches with older monarchs (with more worn wings) should be more likely to harbor infected butterflies. Unfortunately, known pathogen effects on monarch wing characteristics make testing this idea challenging, because patches with more infected monarchs might have higher average wing wear measures simply because parasites negatively affect wing development. Indeed, at the individual level, infected monarchs sampled here had smaller wings and greater wing scale loss (but not greater wing damage); consistent with prior studies showing that infection lowers monarch wing area and body size [30], [31] and reduces the density of black pigmentation on monarch wings [57]. Thus additional studies examining host patch age could provide insight into the potential role of metapopulation ecology in this host-parasite dynamic.

In conclusion, we observed drastically varying prevalence of a protozoan parasite in monarchs inhabiting the Hawaiian Islands, despite high levels of butterfly gene flow and a lack of host population structure. The impact of site-level characteristics and landscape heterogeneity, in addition to colonization-extinction processes, are promising directions that could provide insight into the dynamics of this host-parasite interaction.

## Supporting Information

Figure S1 **Structure plot showing that K (number of distinct populations)  = 1.** Monarchs on the Hawaiian Islands for one admixed genetic population (red text = Big Island, orange text = Maui, blue text = Oahu).
(TIFF)

Table S1 **Field collection site variables.** Latitude and longitude, site type, site area and perimeter (based on the estimated area of the actual plants and not the entire patch), and milkweed species (*Asclepias physocarpa, Calotropis gigantea,* and *Calotropis procera*) were recorded. Only sites with 5 or more monarchs sampled are shown below, as sites with fewer monarchs were excluded from analyses.
(DOCX)

Table S2 **Monarchs used for genetic analysis by sampling site and sampling year.**
(DOCX)

Table S3 **Microsatellite loci used in this study.** Locus name, multiplex reaction, fluorescent label, primer sequences, repeat motif and primer annealing temperature (T_A_). Number of alleles and allele size range were determined by Lyons et al (2012).
(DOCX)

Table S4 **Observed (** ***H_o_*** **) and expected (** ***H_e_*** **) heterozygosity at the seven Hawaiian sites at each locus as calculated by Arlequin 3.5.1.2.**
(DOCX)

Table S5 **Pairwise** ***R*** **_ST_ and** ***F*** **_ST_ values between four islands, as calculated in Arlequin version 3.5.1.2.**
(DOCX)

File S1 **Analysis for site-level characteristics and infection prevalence.**
(DOCX)

## References

[1] Ravensdale M, Nemri A, Thrall PH, Ellis JG, Dodds PN (2011) Co-evolutionary interactions between host resistance and pathogen effector genes in flax rust disease. Molecular Plant Pathology 12: 93–102. 21118351 10.1111/j.1364-3703.2010.00657.x PMC2999005

[2] Ostfeld RS, Glass GE, Keesing F (2005) Spatial epidemiology: an emerging (or re-emerging) discipline. Trends in Ecology & Evolution 20: 328–336. 16701389 10.1016/j.tree.2005.03.009

[3] Smith DL, Lucey B, Waller LA, Childs JE, Real LA (2002) Predicting the spatial dynamics of rabies epidemics on heterogeneous landscapes. Proceedings of the National Academy of Sciences of the United States of America 99: 3668–3672. 11904426 10.1073/pnas.042400799 PMC122581

[4] Allan BF, Keesing F, Ostfeld RS (2003) Effect of forest fragmentation on Lyme disease risk. Conservation Biology 17: 267–272.

[5] Thrall PH, Antonovics J (1995) Theoretical and empirical studies of metapopulations-population and genetic dynamics of the silene-usfilago system. Canadian Journal of Botany-Revue Canadienne De Botanique 73: S1249–S1258.

[6] Thrall PH, Burdon JJ (1997) Host-pathogen dynamics in a metapopulation context: the ecological and evolutionary consequences of being spatial. Journal of Ecology 85: 743–753.

[7] Riley S (2007) Large-scale spatial-transmission models of infectious disease. Science 316: 1298–1301. 17540894 10.1126/science.1134695

[8] Cronin JT (2009) Habitat edges, within-patch dispersion of hosts, and parasitoid oviposition behavior. Ecology 90: 196–207. 19294925 10.1890/08-0208.1

[9] Hassell MP, Comins HN, May RM (1991) Spatial structure and chaos in insect population-dynamics. Nature 353: 255–258.

[10] Altizer S, Bartel R, Han BA (2011) Animal migration and infectious disease risk. Science 331: 296–302. 21252339 10.1126/science.1194694

[11] Johnson TL, Cully JF, Collinge SK, Ray C, Frey CM, et al (2011) Spread of Plague Among Black-Tailed Prairie Dogs Is Associated With Colony Spatial Characteristics. Journal of Wildlife Management 75: 357–368.

[12] Folstad I, Nilssen AC, Halvorsen O, Andersen J (1991) Parasite avoidance: the cause of post-valing migrations in *Rangifer*? Canadian Journal of Zoology 69: 2423–2429.

[13] Bartel RA, Oberhauser KS, de Roode JC, Altizer SM (2011) Monarch butterfly migration and parasite transmission in eastern North America. Ecology 92: 342–351. 21618914 10.1890/10-0489.1 PMC7163749

[14] Carlsson-Graner U (2006) Disease dynamics, host specificity and pathogen persistence in isolated host populations. Oikos 112: 174–184.

[15] Carlsson-Graner U, Thrall PH (2002) The spatial distribution of plant populations, disease dynamics and evolution of resistance. Oikos 97: 97–110.

[16] Best A, Webb S, White A, Boots M (2011) Host resistance and coevolution in spatially structured populations. Proceedings of the Royal Society B-Biological Sciences 278: 2216–2222. 10.1098/rspb.2010.1978 PMC3107622 21147793

[17] Ackery PR, Vane-Wright RI (1984) Milkweed Butterflies: their Cladistics and Biology. Ithaca, NY: Cornell University Press.

[18] Urquhart FA, Urquhart NR (1978) Autumnal migration routes of the eastern population of the monarch butterfly (*Danaus p. plexippus* L.; Danaidae; Lepidoptera) in North America to the overwintering site in the Neovolcanic Plateau of Mexico. Canadian Journal of Zoology 56: 1759–1764.

[19] Brower LP, Malcolm SB (1991) Animal migrations-endangered phenomena. American Zoologist 31: 265–276.

[20] Zalucki MP, Clarke AR (2004) Monarchs across the Pacific: the Columbus hypothesis revisited. Biological Journal of the Linnean Society 82: 111–121.

[21] Shephard JM, Hughes JM, Zalucki MP (2002) Genetic differentiation between Australian and North American populations of the monarch butterfly *Danaus plexippus* (L.) (Lepidoptera: Nymphalidae): an exploration using allozyme electrophoresis. Biological Journal of the Linnean Society 75: 437–452.

[22] Altizer S, Davis AK (2010) Populations of monarch butterflies with different migratory behaviors show divergence in wing morphology. Evolution 64: 1018–1028. 20067519 10.1111/j.1558-5646.2010.00946.x

[23] Lyons JI, Pierce AA, Barribeau SM, Sternberg ED, Mongue AJ, et al (2012) Lack of genetic differentiation between monarch butterflies with divergent migration destinations. Molecular Ecology 21: 3433–3444. 22574833 10.1111/j.1365-294X.2012.05613.x

[24] Altizer S, De Roode JC (2013) Monarch defense against a debilitating parasite: resistance, immunity and self-medication. In: Oberhauser K, Altizer S, Nail KR, editors. Monarchs in a Changing World: Biology and Conservation of an Iconic Insect: Cornell University. pp. *In press*.

[25] Leong KLH, Yoshimura MA, Kaya HK, Williams H (1997) Instar susceptibility of the monarch butterfly (Danaus plexippus) to the neogregarine parasite, Ophryocystis elektroscirrha. Journal of Invertebrate Pathology 69: 79–83. 9028932 10.1006/jipa.1996.4634

[26] Altizer SM, Oberhauser KS, Brower LP (2000) Associations between host migration and the prevalence of a protozoan parasite in natural populations of adult monarch butterflies. Ecological Entomology 25: 125–139.

[27] Leong KLH, Yoshimura MA, Kaya HK (1997) Occurrence of a neogregarine protozoan, Ophryocystis elektroscirrha McLaughlin and Myers, in populations of monarch and queen butterflies. Pan-Pacific Entomologist 73: 49–51.

[28] Altizer SM, Oberhauser K, Geurts KA (2004) Transmission of the protozoan parasite, Ophryocystis elektroscirrha, in monarch butterfly populations. In: Solensky KOaM, editor. The Monarch Butterfly: Biology and Conservation. Ithaca, New York: Cornell University Press. 203–218.

[29] McLaughlin RE, Myers J (1970) *Ophryocystis elektroscirrha* sp. n., a neogregarine pathogen of the monarch butterfly *Danaus plexippus* (L.) and the Florida queen butterfly *D. gilippus berenice* Cramer. Journal of Protozoology 17: 300-&.

[30] de Roode JC, Gold LR, Altizer S (2007) Virulence determinants in a natural butterfly-parasite system. Parasitology 134: 657–668. 17140464 10.1017/S0031182006002009

[31] Altizer SM, Oberhauser KS (1999) Effects of the protozoan parasite Ophryocystis elektroscirrha on the fitness of monarch butterflies (Danaus plexippus). Journal of Invertebrate Pathology 74: 76–88. 10388550 10.1006/jipa.1999.4853

[32] Bradley CA, Altizer S (2005) Parasites hinder monarch butterfly flight: implications for disease spread in migratory hosts. Ecology Letters 8: 290–300.

[33] Coltman DW, Pilkington JG, Smith JA, Pemberton JM (1999) Parasite-mediated selection against inbred Soay sheep in a free-living, island population. Evolution 53: 1259–1267. 28565537 10.1111/j.1558-5646.1999.tb04538.x

[34] Acevedo-Whitehouse K, Petetti L, Duignan P, Castinel A (2009) Hookworm infection, anaemia and genetic variability of the New Zealand sea lion. Proceedings of the Royal Society B-Biological Sciences 276: 3523–3529. 10.1098/rspb.2009.1001 PMC2817199 19605394

[35] Smith EM, Hoffman JI, Green LE, Amos W (2012) Preliminary association of microsatellite heterozygosity with footrot in domestic sheep. Livestock Science 143: 293–299.

[36] Whitehorn PR, Tinsley MC, Brown MJF, Darvill B, Goulson D (2011) Genetic diversity, parasite prevalence and immunity in wild bumblebees. Proceedings of the Royal Society B-Biological Sciences 278: 1195–1202. 10.1098/rspb.2010.1550 PMC3049068 20926436

[37] Zalucki MP, Kitching RL (1982) The analysis and description of movement in adult *Danaus plexippus* L (Lepidotera, Danaiae). Behaviour 80: 174–198.

[38] Cockrell BJ, Malcolm SB, Brower LP (1993) Time, temperature, and latitudinal constraints on the annual recolonization of eastern North America by the monarch butterfly. Natural History Museum of Los Angeles County Science Series 0: 233–251.

[39] de Roode JC, Chi J, Rarick RM, Altizer S (2009) Strength in numbers: high parasite burdens increase transmission of a protozoan parasite of monarch butterflies (Danaus plexippus). Oecologia 161: 67–75. 19418070 10.1007/s00442-009-1361-6

[40] Excoffier L, Lischer HEL (2010) Arlequin suite ver 3.5: a new series of programs to perform population genetics analyses under Linux and Windows. Molecular Ecology Resources 10: 564–567. 21565059 10.1111/j.1755-0998.2010.02847.x

[41] Rice WR (1989) Analyzing tables of statistical tests. Evolution 43: 223–225. 28568501 10.1111/j.1558-5646.1989.tb04220.x

[42] Hood GM (2010) PopTools version 3.2.5.

[43] Rousset F (2008) GENEPOP ‘007: a complete re-implementation of the GENEPOP software for Windows and Linux. Molecular Ecology Resources 8: 103–106. 21585727 10.1111/j.1471-8286.2007.01931.x

[44] Szpiech Z, Jakobsson M, Rosenberg N (2008) ADZE: a rarefaction approach for counting alleles private to combinations of. Bioinformatics 24: 1367–4811. 18779233 10.1093/bioinformatics/btn478 PMC2732282

[45] Pritchard JK, Stephens M, Donnelly P (2000) Inference of population structure using multilocus genotype data. Genetics 155: 945–959. 10835412 10.1093/genetics/155.2.945 PMC1461096

[46] Vane-Wright RI (1993) The Columbus hypothesis: An explantation for the dramatic 19th century range expansion of the monarch butterfly. Natural History Museum of Los Angeles County Science Series 0: 179–187.

[47] Slatkin M (1995) A measure of population subdivision based on microsatellite allele frequencies. Genetics 139: 457–462. 7705646 10.1093/genetics/139.1.457 PMC1206343

[48] Balloux F, Lugon-Moulin N (2002) The estimation of population differentiation with microsatellite markers. Molecular Ecology 11: 155–165. 11856418 10.1046/j.0962-1083.2001.01436.x

[49] Oksanen JF, Blanchet FG, Kindt R, Legendre P, Minchin PR, et al. (2011) vegan: Community Ecology Package. R package version 2.0-0. Available: http://CRANR-projectorg/package=vegan.

[50] Real LA, Childs JE (2005) Spatial-temporal dynamics of rabies in ecological communities. In: Ray SKCaC, editor. Disease Ecology: Community Structure and Pathogen Dynamics. Oxford: Oxford University Press. 168–185.

[51] Almberg ES, Cross PC, Smith DW (2010) Persistence of canine distemper virus in the Greater Yellowstone Ecosystem’s carnivore community. Ecological Applications 20: 2058–2074. 21049890 10.1890/09-1225.1

[52] Hess G, Randolph S, Arneberg P, Chemini C, Furlanello C, et al.. (2002) Spatial aspects of disease dynamics. In: P. Hudson AR, B. T Grenfell, H Heesterbeek, and A. P Dobson, editor. Ecology of Wildlife Diseases. Oxford: Oxford University Press. 102–119.

[53] Hanski I, Gilpin M (1991) Metapopulation dynamics- brief history and conceptual domain. Biological Journal of the Linnean Society 42: 3–16.

[54] Hess G (1996) Disease in metapopulation models: Implications for conservation. Ecology 77: 1617–1632.

[55] McCallum H, Dobson A (2002) Disease, habitat fragmentation and conservation. Proceedings of the Royal Society of London Series B-Biological Sciences 269: 2041–2049. 10.1098/rspb.2002.2079 PMC1691124 12396504

[56] Gog J, Woodroffe R, Swinton J (2002) Disease in endangered metapopulations: the importance of alternative hosts. Proceedings of the Royal Society B-Biological Sciences 269: 671–676. 10.1098/rspb.2001.1667 PMC1690941 11934357

[57] Lindsey E, Altizer S (2009) Sex differences in immune defenses and response to parasitism in monarch butterflies. Evolutionary Ecology 23: 607–620.

