# Supplementary figures and images for: Extreme Heterogeneity in Parasitism Despite Low Population Genetic Structure among Monarch Butterflies Inhabiting the Hawaiian Islands
Source: PLoS One. 2014 Jun 13;9(6):e100061. doi: 10.1371/journal.pone.0100061 (PMC4057267; doi:10.1371/journal.pone.0100061)

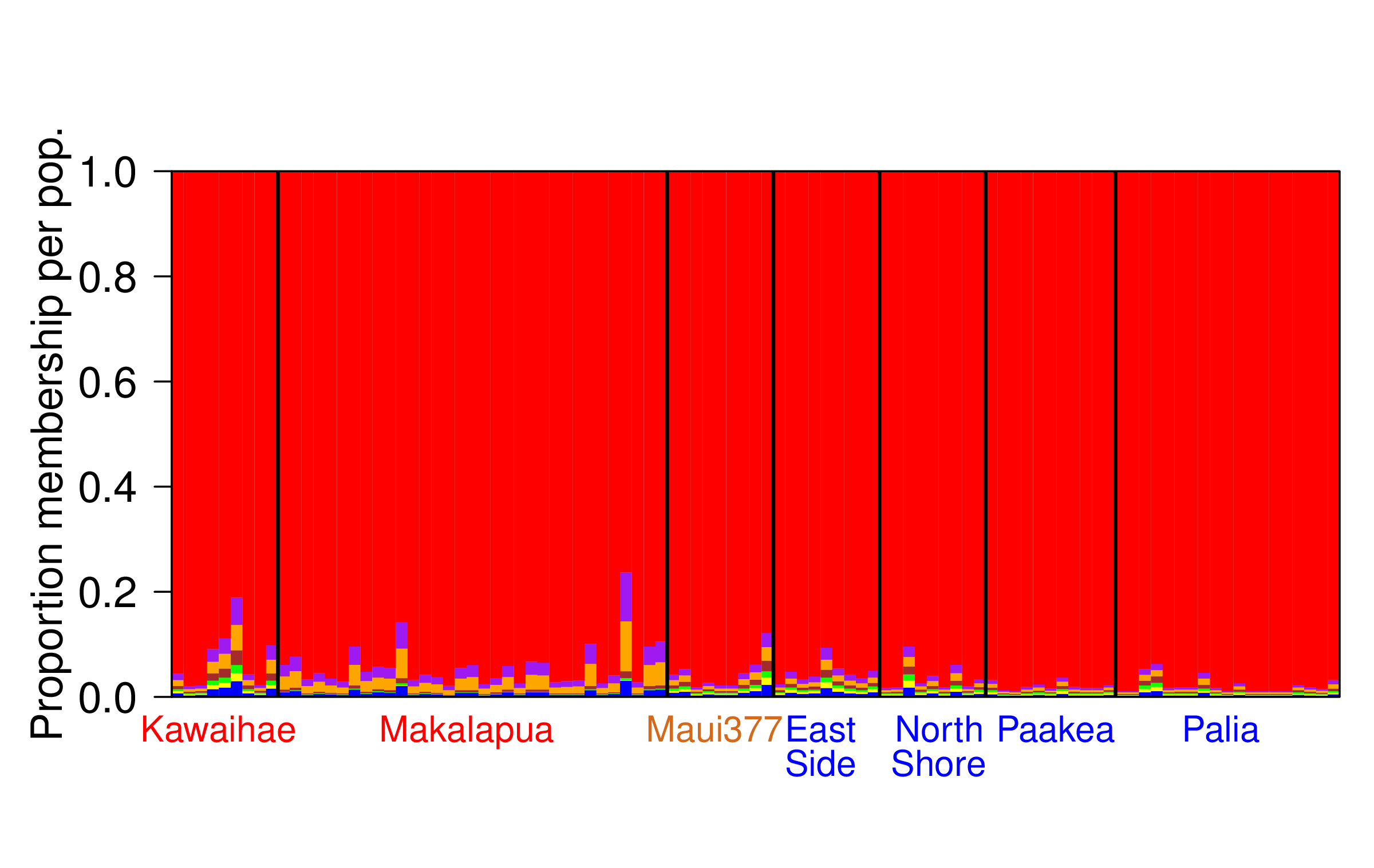

Supplement: Figure S1 — Structure plot showing that K (number of distinct populations) = 1. Monarchs on the Hawaiian Islands for one admixed genetic population (red text = Big Island, orange text = Maui, blue text = Oahu). (TIFF) [file pone.0100061.s001.tiff]
