# Supplementary material for: Extreme Heterogeneity in Parasitism Despite Low Population Genetic Structure among Monarch Butterflies Inhabiting the Hawaiian Islands
Source: PLoS One. 2014 Jun 13;9(6):e100061. doi: 10.1371/journal.pone.0100061 (PMC4057267; doi:10.1371/journal.pone.0100061)
Supplement: Table S1 — Field collection site variables. Latitude and longitude, site type, site area and perimeter (based on the estimated area of the actual plants and not the entire patch), and milkweed species (Asclepias physocarpa, Calotropis gigantea, and Calotropis procera) were recorded. Only sites with 5 or more monarchs sampled are shown below, as sites with fewer monarchs were excluded from analyses. (DOCX) [file pone.0100061.s002.docx]

**Table S1.** Field collection site variables, including site latitude and longitude, site type, site area and perimeter (based on the estimated area of the actual plants and not the entire patch), and milkweed species (*Asclepias physocarpa, Calotropis gigantea,* and *Calotropis procera*) were recorded. Only sites with 5 or more monarchs sampled are shown below, as sites with fewer monarchs were excluded from analyses.

| **Site** | **Island** | **Latitude** | **Longitude** | **Site Type** | **Area (m^2^)** | **Perimeter (m)** | **Milkweed Species** |
| --- | --- | --- | --- | --- | --- | --- | --- |
| Kailua-Kona | Big Island | 19.64 | -155.99 | Urban | 2535.78 | 203.35 | *C. gigantea* |
| Kawaihae | Big Island | 20.04 | -155.82 | Rural | 15352.53 | 575.54 | *C. gigantea* |
| Konacopia Farms | Big Island | 19.46 | -155.89 | Rural | 817.71 | 145.91 | *C. gigantea* |
| Makalapua | Big Island | 19.65 | -156.00 | Urban | 297.15 | 119.17 | *C. gigantea* |
| Kealia Beach | Kauai | 22.09 | -159.31 | Rural | 14903.32 | 496.41 | *C. gigantea* |
| Kekaha Beach | Kauai | 21.97 | -159.73 | Suburban | 918.60 | 156.50 | *C. procera* |
| Waimea | Kauai | 21.96 | -159.67 | Urban | 103623.17 | 1382.26 | *C. gigantea* |
| West Waimea | Kauai | 21.96 | -159.69 | Suburban | 84.26 | 37.14 | *C. procera* |
| Kihei | Maui | 20.73 | -156.45 | Suburban | 365.35 | 74.91 | *C. gigantea* |
| Maui 377 | Maui | 20.83 | -156.32 | Rural | 396.98 | 76.22 | *A. physocarpa* |
| Maui-Lani | Maui | 20.86 | -156.48 | Suburban | 46.42 | 27.42 | *C. procera* |
| East Side | Oahu | 21.55 | -157.85 | Suburban | 488.42 | 105.42 | *C. gigantea* |
| Nehoa St. | Oahu | 21.31 | -157.83 | Suburban | 758.39 | 111.76 | *C. gigantea* |
| North Shore | Oahu | 21.68 | -158.03 | Suburban | 56168.23 | 1034.63 | *C. gigantea* |
| Palia St. | Oahu | 21.40 | -158.02 | Suburban | 225.27 | 60.91 | *C. gigantea* |
| Paakea Rd. | Oahu | 21.45 | -158.18 | Suburban | 215 | 45.25 | *C. gigantea* |
