# Supplementary material for: Extreme Heterogeneity in Parasitism Despite Low Population Genetic Structure among Monarch Butterflies Inhabiting the Hawaiian Islands
Source: PLoS One. 2014 Jun 13;9(6):e100061. doi: 10.1371/journal.pone.0100061 (PMC4057267; doi:10.1371/journal.pone.0100061)
Supplement: Table S2 — Monarchs used for genetic analysis by sampling site and sampling year. (DOCX) [file pone.0100061.s003.docx]

**Table S2.** Monarchs used for genetic analysis by sampling site and sampling year.

|  | **2007** | **2009** | **2010** | **Total** | **Proportion Infected** |
| --- | --- | --- | --- | --- | --- |
| **Big Island** |  |  |  |  |  |
| Kawaihae |  |  | 9 | 9 | 0.11 |
| Makalapua | 11 | 5 | 17 | 33 | 0.55 |
| **Oahu** |  |  |  |  |  |
| East Side |  |  | 9 | 9 | 0.11 |
| North Shore |  |  | 9 | 9 | 0.33 |
| Paakea | 11 |  |  | 11 | 0.00 |
| Palia | 7 | 7 | 5 | 19 | 0.63 |
| **Maui** |  |  |  |  |  |
| 377 |  |  | 9 | 9 | 0.00 |
