# Supplementary material for: Extreme Heterogeneity in Parasitism Despite Low Population Genetic Structure among Monarch Butterflies Inhabiting the Hawaiian Islands
Source: PLoS One. 2014 Jun 13;9(6):e100061. doi: 10.1371/journal.pone.0100061 (PMC4057267; doi:10.1371/journal.pone.0100061)
Supplement: Table S3 — Microsatellite loci used in this study. Locus name, multiplex reaction, fluorescent label, primer sequences, repeat motif and primer annealing temperature (TA). Number of alleles and allele size range were determined by Lyons et al (2012). (DOCX) [file pone.0100061.s004.docx]

**Table S3.** Microsatellite loci used in this study, showing locus name, multiplex reaction, fluorescent label, primer sequences, repeat motif and primer annealing temperature (T_A_). Number of alleles and allele size range were determined by Lyons et al (2012).

| **Locus** | **Multiplex reaction** | **Label** | **Primer sequence** | **Repeat motif** | **T_A_** | **No. alleles** | **Allele size range** |
| --- | --- | --- | --- | --- | --- | --- | --- |
| **168** | 1 | FAM | F: AGTTCAGGGTTTACGTGAGCA | tcata | 57°C | 6 | 143-168 |
|  |  |  | R: CATTATGTGAAGTGTTGCATGG |  |  |  |  |
| **153** | 1 | FAM | F: TGCGAAAASTGGTTTGAGGT | ta | 57°C | 10 | 228-258 |
|  |  |  | R: TTATCGCCAAGTAAGTAATTTCG |  |  |  |  |
| **320** | 2 | HEX | F: AATTTCTTGAGCGCTTTATCC | at | 57°C | 18 | 153-187 |
|  |  |  | R: CTGATCCTCGTCATCTCTCG |  |  |  |  |
| **197** | 2 | FAM | F: TGTCATTTCGATGTCGGCTA | att | 57°C | 4 | 174-183 |
|  |  |  | R: CAGAGAGAGCCTCGGGTAAA |  |  |  |  |
| **208** | 3 | FAM | F: TTTAGGACCCCAATCGGATTTTCG | at | 60°C | 19 | 178-242 |
|  |  |  | R: CGCGGACATTTTCACTTTCACGAT |  |  |  |  |
| **203** | 3 | HEX | F: TGACATACTTTATGTTCGTGGAAGG | at | 60°C | 14 | 196-222 |
|  |  |  | R: CCGCTCGCCTATATACAGGACACA |  |  |  |  |
| **141** | 4 | FAM | F: TCAAACCCGCATCCCTAGTGGTA | tc | 60°C | 13 | 150-178 |
|  |  |  | R: TGGCAACGTACAGGGACGTGA |  |  |  |  |
| **1679** | 4 | FAM | F: ATAGCCCTTCGACTTGTCGTTTCTC | tat | 60°C | 4 | 215-224 |
|  |  |  | R:TCGACTGATGTTTTCgGGACTacGA |  |  |  |  |
| **137** | 5 | HEX | F: AAGGTGGCGGTAAAAAGGCACAGA | aag | 60°C | 3 | 239-248 |
|  |  |  | R: TCGCTTTCTTCCTCTTCCTCCTCA |  |  |  |  |
| **122** | 5 | FAM | F: TTATAAGACCTCAACACCCACGAA | tta | 60°C | 6 | 228-252 |
|  |  |  | R: CGCCGCTTCTAAATGAGTGGGATT |  |  |  |  |
| **494** | 6 | HEX | F: CCGCGCTAGTCATTGTGTGAATGT | att | 60°C | 7 | 160-181 |
|  |  |  | R: CCTCGACTGATAGCCTTCGAAACG |  |  |  |  |
| **983** | 6 | FAM | F:AGACGCTTtGTTCAGCTTCGACCAC | ac | 60°C | 15 | 223-257 |
|  |  |  | R: ttTaCGaTCACTCATACgaAACGGTa |  |  |  |  |
| **854** | 8 | HEX | F:AACGTCATCTGCACACGCCATACTA | at | 67°C | 8 | 230-254 |
|  |  |  | R:TCCAATTAAACGTGACGCCATTTTG |  |  |  |  |
| **165** | 8 | FAM | F:CCTCcGGAACCTGTCAAGAAAAaGA | tat | 67°C | 8 | 189-213 |
|  |  |  | R:CACTCATCAGAACTGAAAAGTTCGAGACC |  |  |  |  |
| **819** | 8 | FAM | F:GACTCGGAGACATGAGATCGACGAC | cacga | 67°C | 11 | 213-263 |
|  |  |  | R:TCGTCAGACAATTGCTCAAAATGGA |  |  |  |  |
| **519** | 9 | FAM | F:GTGGCGGGGCTTTGTGTAAATAAGA | att | 63°C | 15 | 221-263 |
|  |  |  | R:CAGGGTTCCATACAAACGTGTGATACAATA |  |  |  |  |
