# Supplementary material for: Extreme Heterogeneity in Parasitism Despite Low Population Genetic Structure among Monarch Butterflies Inhabiting the Hawaiian Islands
Source: PLoS One. 2014 Jun 13;9(6):e100061. doi: 10.1371/journal.pone.0100061 (PMC4057267; doi:10.1371/journal.pone.0100061)
Supplement: Table S4 — Observed ( Ho ) and expected ( He ) heterozygosity at the seven Hawaiian sites at each locus as calculated by Arlequin 3.5.1.2. (DOCX) [file pone.0100061.s005.docx]

**Table S4.** Observed (*H_o_*) and expected (*H_e_*) heterozygosity at the seven Hawaiian sites at each locus as calculated by Arlequin 3.5.1.2

|  | Kawaihae | | Makalapua | | Maui 377 | | EastSide | | NorthShore | | Paakea | | Palia | |
| --- | --- | --- | --- | --- | --- | --- | --- | --- | --- | --- | --- | --- | --- | --- |
| Locus | *H_0_* | *H_e_* | *H_0_* | *H_e_* | *H_0_* | *H_e_* | *H_0_* | *H_e_* | *H_0_* | *H_e_* | *H_0_* | *H_e_* | *H_0_* | *H_e_* |
| 168 | 0.778 | 0.542 | 0.424 | 0.516 | 0.778 | 0.529 | 0.667 | 0.582 | 0.889 | 0.739 | 0.909 | 0.671 | 0.474 | 0.650 |
| 153 | 0.667 | 0.627 | 0.364 | 0.553 | 0.333 | 0.386 | 0.444 | 0.627 | 0.333 | 0.464 | 0.273 | 0.385 | 0.053 | 0.235 |
| 320 | 0.444 | 0.673 | 0.667 | 0.723 | 0.222 | 0.627* | 0.667 | 0.712 | 0.667 | 0.660 | 0.364 | 0.671 | 0.556 | 0.641* |
| 197 | 0.444 | 0.471 | 0.424 | 0.429 | 0.333 | 0.307 | 0.444 | 0.471 | 0.444 | 0.366 | 0.455 | 0.368 | 0.222 | 0.203 |
| 208 | 0.444 | 0.542 | 0.438 | 0.546 | 0.556 | 0.569 | 0.778 | 0.699 | 0.556 | 0.699 | 0.455 | 0.541 | 0.211 | 0.568 |
| 203 | 0.444 | 0.529 | 0.594 | 0.604 | 0.556 | 0.712 | 0.556 | 0.634 | 0.444 | 0.752 | 0.364 | 0.498 | 0.789 | 0.596 |
| 141 | 0.444 | 0.556 | 0.485 | 0.502 | 0.444 | 0.399 | 0.778 | 0.778 | 0.889 | 0.667 | 0.455 | 0.593 | 0.474 | 0.538 |
| 1679 | 0.222 | 0.471 | 0.545 | 0.516 | 0.222 | 0.209 | 0.222 | 0.582 | 0.333 | 0.425 | 0.545 | 0.589 | 0.474 | 0.494 |
| 137 | - | - | - | - | - | - | - | - | - | - | - | - | - | - |
| 122 | 0.333 | 0.294 | 0.061 | 0.060 | - | - | 0.222 | 0.209 | 0.111 | 0.111 | - | - | - | - |
| 494 | 0.333 | 0.294 | 0.030 | 0.030 | 0.222 | 0.209 | 0.111 | 0.111 | - | - | - | - | - | - |
| 983 | 0.222 | 0.209 | 0.242 | 0.322 | 0.111 | 0.503 | 0.556 | 0.503 | 0.333 | 0.425 | 0.545 | 0.610 | 0.261 | 0.627 |
| 854 | 0.333 | 0.503 | 0.303 | 0.505* | 0.444 | 0.399 | 0.333 | 0.425 | 0.000 | 0.366 | 0.300 | 0.268 | 0.389 | 0.417 |
| 165 | 0.111 | 0.307 | 0.273 | 0.373 | 0.333 | 0.542 | 0.222 | 0.366 | 0.333 | 0.503 | 0.400 | 0.505 | 0.167 | 0.475 |
| 819 | 0.778 | 0.569 | 0.727 | 0.669 | 0.444 | 0.569 | 0.444 | 0.503 | 0.667 | 0.627 | 0.700 | 0.595 | 0.500 | 0.422 |
| 519 | 0.667 | 0.719 | 0.636 | 0.729 | 0.667 | 0.654 | 0.667 | 0.582 | 0.667 | 0.569 | 0.545 | 0.515 | 0.421 | 0.632 |

Loci for which observed and expected heterozygosities are significantly different are indicated with asterisks; significance was determined using an α of 0.05 and a sequential Bonferroni correction. Loci for which at least four populations were in Hardy-Weinberg equilibirum and which were polymorphic were used for subsequent analyses and are indicated in shading. Dashes indicate monomorphic loci.
