# Supplementary material for: Extreme Heterogeneity in Parasitism Despite Low Population Genetic Structure among Monarch Butterflies Inhabiting the Hawaiian Islands
Source: PLoS One. 2014 Jun 13;9(6):e100061. doi: 10.1371/journal.pone.0100061 (PMC4057267; doi:10.1371/journal.pone.0100061)
Supplement: Table S5 — Pairwise R ST and F ST values between four islands, as calculated in Arlequin version 3.5.1.2. (DOCX) [file pone.0100061.s006.docx]

**Table S5.** Pairwise *R*_ST_ and *F*_ST_ values between four islands, as calculated in Arlequin version 3.5.1.2

|  | **Big Island** | **Kauai** | **Maui** |
| --- | --- | --- | --- |
| **Kauai** | *R*_ST_: 0.00000 |  |  |
|  | *F*_ST_: 0.02133* |  |  |
| **Maui** | *R*_ST_: 0.01155 | *R*_ST_: 0.00000 |  |
|  | *F*_ST_: 0.03755* | *F*_ST_: 0.00593 |  |
| **Oahu** | *R*_ST_: 0.02054* | *R*_ST_: 0.00000 | *R*_ST_: 0.00165 |
|  | *F*_ST_: 0.04943* | *F*_ST_: 0.01376* | *F*_ST_: 0.00729 |

Asterisks and shading denote values that are significantly different from zero.
