# Supplementary material for: Extreme Heterogeneity in Parasitism Despite Low Population Genetic Structure among Monarch Butterflies Inhabiting the Hawaiian Islands
Source: PLoS One. 2014 Jun 13;9(6):e100061. doi: 10.1371/journal.pone.0100061 (PMC4057267; doi:10.1371/journal.pone.0100061)
Supplement: File S1 — Analysis for site-level characteristics and infection prevalence. (DOCX) [file pone.0100061.s007.docx]

**Analysis for site-level characteristics and infection prevalence.** To test whether site-level variables predicted infection prevalence, we calculated average prevalence per site per year, and used arcsin-square root-transformed values (to normalize the error variance) as the dependent variable in an analysis of variance. The following main effects were included in the model: Island, Year, Host Plant Species and Patch Type (treated as categorical variables), and Log_10_-transformed Patch Area (treated as a continuous covariate). Each site was assigned a Patch Type (urban, suburban, rural) based on qualitative assessment of distance to nearest city, impervious surface and human activity. We measured Patch Area as the area of coverage of milkweed plants per site in m^2^. Host plant species was represented per site as presented in Table S1. The analysis was weighted using sample size per year-site combination (to reflect better confidence in samples for which more monarchs were tested). Results showed that infection prevalence was not predicted by any independent variable included in the multivariate model, with significance tests as follows: LogArea (F _1,17_ = 2.90; P = 0.11); Island (F _3,17_ = 1.52; P = 0.25); Year (F _2,17_ = 1.04; P = 0.38); HostPlant (F _2,17_ = 2.58; P = 0.11); PatchType (F _2,17_ = 1.52; P = 0.25). The lack of significant effects can partly be explained by limited statistical power from low sample size (N = 28 site by year combinations) and by the high variation in prevalence among sites relative to any mean differences in levels of each independent variable.
